# Knock down of Whitefly Gut Gene Expression and Mortality by Orally Delivered Gut Gene-Specific dsRNAs

**DOI:** 10.1371/journal.pone.0168921

**Published:** 2017-01-03

**Authors:** Meenal Vyas, Amir Raza, Muhammad Yousaf Ali, Muhammad Aleem Ashraf, Shahid Mansoor, Ahmad Ali Shahid, Judith K. Brown

**Affiliations:** 1 School of Plant Sciences, University of Arizona, Tucson, Arizona, United States of America; 2 National Institute for Biotechnology & Genetic Engineering (NIBGE), Faisalabad, Pakistan; 3 Centre of Excellence in Molecular Biology (CEMB), University of Punjab, Lahore, Pakistan; Chinese Academy of Agricultural Sciences, CHINA

## Abstract

Control of the whitefly *Bemisia tabaci* (Genn.) agricultural pest and plant virus vector relies on the use of chemical insecticides. RNA-interference (RNAi) is a homology-dependent innate immune response in eukaryotes, including insects, which results in degradation of the corresponding transcript following its recognition by a double-stranded RNA (dsRNA) that shares 100% sequence homology. In this study, six whitefly ‘gut’ genes were selected from an *in silico*-annotated transcriptome library constructed from the whitefly alimentary canal or ‘gut’ of the B biotype of *B*. *tabaci*, and tested for knock down efficacy, post-ingestion of dsRNAs that share 100% sequence homology to each respective gene target. Candidate genes were: *Acetylcholine receptor subunit α*, *Alpha glucosidase 1*, *Aquaporin 1*, *Heat shock protein 70*, *Trehalase1*, and *Trehalose transporter1*. The efficacy of RNAi knock down was further tested in a gene-specific functional bioassay, and mortality was recorded in 24 hr intervals, six days, post-treatment. Based on qPCR analysis, all six genes tested showed significantly reduced gene expression. Moderate-to-high whitefly mortality was associated with the down-regulation of osmoregulation, sugar metabolism and sugar transport-associated genes, demonstrating that whitefly survivability was linked with RNAi results. Silenced *Acetylcholine receptor subunit α* and *Heat shock protein 70* genes showed an initial low whitefly mortality, however, following insecticide or high temperature treatments, respectively, significantly increased knockdown efficacy and death was observed, indicating enhanced post-knockdown sensitivity perhaps related to systemic silencing. The oral delivery of gut-specific dsRNAs, when combined with qPCR analysis of gene expression and a corresponding gene-specific bioassay that relates knockdown and mortality, offers a viable approach for functional genomics analysis and the discovery of prospective dsRNA biopesticide targets. The approach can be applied to functional genomics analyses to facilitate, species-specific dsRNA-mediated control of other non-model hemipterans.

## Introduction

The whitefly *Bemisia tabaci* (Genn.) sibling species group (*Hemiptera*, *Aleyrodidae*) comprises an unknown number of cryptic species [1,2]. Initially, several well-studied cryptic species were referred to as ‘biological types’, conveying an early recognition that the group comprises distinct phenotypes e.g. host range, dispersal behavior, fecundity, virus transmission competency, and insecticide resistance [3,4]. It is distributed in tropical, subtropical and temperate locales, worldwide, that experience mild winters. Several ‘biotypes’ have become recognized as agricultural pests that cause extensive damage to high-density, monoculture crops due to feeding and diseases caused by the plant viruses they transmit, however, many cryptic species are benign in their native habitat [1,2,4,5]. With the recent expansion of controlled-environment vegetable production and the widespread transport of vegetable seedlings and ornamental plants [1,2,5], several exotic *B*. *tabaci* have been introduced. Among the most problematic biotypes have been the B and Q (mitochondria-type, or ‘haplotype’) (1–3), the former, also referred to as *B*. *argentifolli* [4], and the former and latter, also referred to as MEAM I and MED), respectively [3], have become established in agricultural systems, worldwide. In monoculture settings *B*. *tabaci* has been difficult to control for a number of reasons, including that they have high fecundity, often have a broad host range, and have a propensity to develop insecticide resistance [1,2,4,5, 6–11]. Consequently, effective control of *B*. *tabaci* in multiple crop species requires the availability of insecticides with different modes of action to minimize development of insecticide resistance, while also being mindful of environmental considerations [1,2,10].

RNA interference (RNAi) is an anti-viral mechanism that occurs naturally in multicellular organisms that leads to activation of defense response, which recognize the corresponding homologous, double-stranded RNA (dsRNA) and targets it for enzymatic degradation. The role of dsRNA as the initiator molecule for RNAi in animals was first discovered in the nematode *Caenorhabditis elegans* [12]. Knowledge that RNAi pathways occur ubiquitously across higher organisms, has led to the exploitation of dsRNA-mediated functional genomics analysis to experimentally induce gene silencing.

Different mechanisms are operational in dsRNA-mediated gene silencing in insects, and they can involve cell-autonomous and/or systemic silencing pathways. Functional genomics studies have been feasible for a number of model insects, owing to the availability of a genome sequence. However, only recently have transcriptome and/or genome sequences been determined for a number of non-model insects, making functional genomics studies possible across other insect families, in particular, by exploiting species-specific strategies that rely on unique gene regions, and/or on gene sequences conserved across insect families and orders, or on highly conserved, predicted functional domains [13].

Synthetic dsRNA molecules have been delivered to insects by different approaches [13,14–20]. Microinjection of a synthetic dsRNA solution into the hemolymph has been demonstrated to induce RNAi in hemipterans, such as whitefly (*B*. *tabaci)* [21], pea aphid (*Acyrthosiphon pisum*) [22,23], glassy winged sharpshooter (*Homalodiscus vitripennis*) [24], and for dipterans, such as the fruit fly (*Drosophila melanogaster*) [25]. Initially, microinjection was the prefered mode of delivery because it was not known whether RNAi was cell-autonomous and/or systemic among taxonomically different groups of insect groups [12,26–28].

Recently, gene silencing has been demonstrated for non-model insects following oral delivery of synthetic dsRNAs dissolved in 15–30% sucrose, ingested through an artificial membrane [20, 29–32]. This is possible because both cell-autonomous and systemic silencing machinery are known to occur in hemipterans, including the whitefly *B*. *tabaci* [29–31], pea aphid *A*. *pisum* (L.), [20] and potato psyllid *Bactericera cockerelli* (Sulc.) [32]. For phloem-feeding insects, knock down of genes expressed in the alimentary canal is strategic, because the first point of contact for plant sap entering the insect during feeding is the gut. Thus, there is a great interest in functional genomics analysis of hemipteran gut genes as promising biopesticidal targets. Further, delivery of synthetic dsRNAs directly to the gut allows for quantification of dsRNA knockdown efficacy for both ‘within-replicate’ and ‘between-gene’ comparisons for individual insects.

Although oral delivery and microinjection of dsRNAs are ideal for laboratory studies, they are not scalable to field use [33–35]. Recently, gene silencing by the foliar application of synthetic dsRNAs was successful for controlling a chewing insect of grapevines [36], and in another study system, dsRNA was delivered in bait to achieve control of a viral pathogen of the honey bee *Apis mellifera* (L.) [37]. Consequently, there is increased optimism in using RNAi to control phytophagous insect pests and pathogens of insects using dsRNA by various delivery modes including in bait, and by soil drench, stem or trunk injection, or foliar application.

Insect gut genes are expected to be among the most vulnerable knock down targets because they encode proteins involved in essential physiological functions. Traditional pesticides most often disrupt electrochemical signals essential for insect neurotransmission, causing mortality by altering normal responses to environmental stimuli. For example, the nicotinic acetylcholine receptors (nAChRs) are required for insect cognition and behavior [38], and historically have been effective targets for insect control. Osmotic regulation within the gut is essential for survival of phloem-feeders because they ingest large amounts of sap that contains in addition to water and amino acids, sugars, that contribute to an osmotic potential that is five times greater than that of the insect gut [39]. To circumvent this caveat, sugars are hydrolyzed and/or transported from the gut into the hemolymph, and excreted as honeydew [40]. This is accomplished by *Alpha glucosidase 1* (*AGLU1*) and *Aquaporin 1* (*AQP1*), respectively, gut proteins that enzymatically cleave the sugars and transport the breakdown products out of the gut, respectively [39, 41–44].

As a cryptic species, *B*.*tabaci* inhabit a wide variety of climatic environments [10] and due to their small size they are susceptible to desiccation, which is countered in part by waxes applied to body surfaces [45–46], together with up-regulation of heat shock proteins (HSPs) [47,48]. In addition, whitefly-encoded transporter proteins and certain enzymes metabolize and transport sugars that help combat heat stress. The non-reducing disaccharide, trehalose (*α-*D-glucopyranosyl *α-*D glucopyranoside) [49,50], is ubiquitous in insects, microorganisms, and plants, but absent in mammals [51]. It is hydrolyzed into two glucose molecules by *trehalase 1* (*Tre1*) [52], and the glucose is used to provide energy during flight [53], for chitin synthesis [54], and to combat cold [55]. In these capacities, the expression of *trehalose transporter 1* (*Tret1*) is upregulated [56] to promote trehalose accumulation. Finally, whitefly fecundity is influenced by host plant quality and temperature [57–60], with faster development at higher over lower temperatures e.g at 28°C, a life cycle is completed in 17–18 days [58–61].

In this study, transcripts of the ‘B’ biotype of *B tabaci* were mined from an *in silico*-annotated gut transcript library (authors, unpublished), and functionally characterized using reverse genetics by induction gene silencing in whitefly adults, post-ingestion of dsRNAs sharing 100% sequence homology with the respective gene. The selection of gene targets was based on their predicted role in neurotransmission, osmoregulation, thermal tolerance, sugar metabolism and sugar transport, and the *a priori* knowledge that the gene is considered essential for whitefly survival. Gene expression was quantified using quantitative polymerase chain reaction (qPCR) amplification. The knock down efficacy of dsRNA was challenged in bioassays designed to specifically challenge the functional integrity of each gene, respectively, documented as mortality at 24-hour intervals post-treatment, for six days. Results based on reduced mRNA levels, indicated that gene expression for all six gut gene targets was down-regulated, leading to increased mortality. It further illustrates the potential of dsRNA as an organ-directed, species-specific bio-pesticide for whitefly control.

## Materials and Methods

### Whitefly colony maintenance

The whitefly, *B*. *tabaci*, Arizona B biotype (AZ-B) [5] colony was established in the laboratory collected in 1988 after its discovery on commercial poinsettia plants in Tucson, Arizona. Thereafter, it was maintained on cotton or pumpkin plants by serial transfer, approximately every four weeks. For this study, whiteflies were reared on cotton (*Gossypium hirsutum cv* Deltapine 5415) plants infested at the 8–10 leaf stage, maintained in an insect-proof cage (60”x60”x60”cm; BugDorm Store, MegaView Science Co., Ltd. Taiwan) in an otherwise insect-free room maintained at 26±1°C with a relative humidity of 60–70% and 14:10 h light and dark period. Identification of AZ-B was first based on its unique ‘B’ type esterase pattern [5], and thereafter, on DNA sequencing of a 780 base pair (bp) mitochondrial cytochrome oxidase I gene (mtCOI) [5] fragment that when analyzed by phylogenetic analysis with geographically represented *B*. *tabaci* references, revealed that AZ-B biotype was endemic to the North African-Mediterranean-Middle Eastern region (NA-MED-ME) [1,61].

### Candidate gene selection and validation

Functional genomics was carried out for whitefly gut-specific genes with predicted involvement in neurotransmission [38], osmoregulation [39–40], thermal tolerance [47,48] and sugar metabolism/transport [49,50,and 56]. The DNA (gene) or mRNA amplicons were obtained by PCR or reverse transcriptase PCR (RT-PCR) amplification, respectively (Table 1), cloned and the DNA sequences determined, using standard molecular biology methods (http://molecularcloning.com/index.php?prt=31). Amplification was carried out using JumpStart^™^ REDTaq^®^ ReadyMix^™^ PCR reaction mix (SIGMA-ALDRICH, St. Louis, USA) and cycling parameters were 94°C for 3 min for 1 cycle, followed by 35 cycles of denaturation at 94°C for 30 sec, annealing at 55°C for 30 sec, and amplification at 72°C for 45 sec, with a final extension cycle at 72°C for 10 min. RT PCR reactions were carried out using Superscript^®^ III One-Step RT-PCR System with Platinum^®^ Taq DNA Polymerase (Invitrogen, USA). The cDNA synthesis employed an initial denaturation at 94°C for 2 min followed by incubation at 55°C for 30 sec. The cyclic conditions were: initial denaturation at 94°C, for 2min, and then 40 cycles of denaturation at 94°C, 15sec, annealing at 58°C for 30sec, and extension at 68°C, 1 min, with a final 5 min extension step at 68°C. The amplicons were cloned into the pGEM-T^®^ Easy plasmid vector (Promega, USA), according to the manufacturer’s instructions, and subjected to bi-directional, Sanger capillary sequencing at the University of Arizona Genomics Core (UAGC) facility. The sequences were subjected to BLASTn analysis against arthropod and human sequences in the GenBank database (http://www.ncbi.nlm.nih.gov/genbank) to rule out potential for unintended homology between them and the whitefly sequence targets selected for dsRNA design. No matches greater than 3–6 contiguous bases were identified.

**Table 1:** Primers used for PCR and RT-PCR amplification and *in vitro* transcription. The T7 promoter sequence is underlined.

Gene name	Forward primer	Reverse Primer
***AChRα***	TAATACGACTCACTATAGGGAGACCAC TCTCATGTTGCGGGATGTTA	TAATACGACTCACTATAGGGAGACCAC CCTGAAATGCTCGTCCTCTC
***AGLU1***	TAATACGACTCACTATAGGGAGACCAC CCTGGATTGCCTTTTGGTAA	TAATACGACTCACTATAGGGAGACCAC AATGGCGAGACCAAGAATTG
***AQP1***	TAATACGACTCACTATAGGGAGACCAC GAGCCATCTGTGGAGCAATC	TAATACGACTCACTATAGGGAGACCAC AGCAATTGCGAATCCTATCG
***Hsp70***	TAATACGACTCACTATAGGGAGACCAC GCTCACCAAGCAAATGATTCT	TAATACGACTCACTATAGGGAGACCAC GCTGCATCACTGAGTGGAAA
***Tre1***	TAATACGACTCACTATAGGGAGACCAC AAATCTCCCGGTTCTGGAGT	TAATACGACTCACTATAGGGAGACCAC CAGGGTGAAATCCTCCTTGA
***Tret1***	TAATACGACTCACTATAGGGAGACCAC ATTCCACTCTTGGCCATCTG	TAATACGACTCACTATAGGGAGACCAC ATCTCCTCGTTGACCACCTG
***rsGFP***	TAATACGACTCACTATAGGGAGACCAC TCAGTGGAGAGGGTGAAGGT	TAATACGACTCACTATAGGGAGACCAC GATCCTGTTGACGAGGGTGT

One sequence representing each cloned whitefly gene/mRNA was submitted to the NCBI-GenBank database and assigned the following Accession numbers: *Acetylcholine receptor subunit α* (*AChRα*) (KF377802.1); *Alpha glucosidase 1* (*AGLU1*) (KF377803.1); *Aquaporin 1* (*AQP1*) (KF377800.1); *Heat shock protein 70* (*Hsp70*) (KF377804.1); *Trehalase1* (*Tre1*) (KF442965.1); and *Trehalose transporter-1* (*Tret-1*) (KF442966.1).

### RNA interference

#### *In vitro* transcription

The T7 promoter [21] was incorporated into the 5’-end of the forward and reverse primers (Table 1) to enable *in vitro* transcription. The dsRNA corresponding to each gene was synthesized using MEGA script^®^ RNAi kit (Ambion, USA), and amplicons purified from a 1.2% agarose gel were used as template for *in vitro* transcription (Ambion, USA). The resultant dsRNA was purified and quantified using a Nanodrop2000 spectrophotometer (Thermo Scientific Inc., USA), and stored at -20°C until used for oral delivery.

#### Whitefly oral delivery of *in vitro* synthesized dsRNA

For each gene target, experiments consisted of two biological replicates, with two technical replicates each. Adult whiteflies (200) were collected from the AZ-B biotype colony [5] using a hand-held aspirator, and transferred to a glass vial covered with Parafilm M^®^ (Denville Scientific Inc., USA). The dsRNA was added to sterile 20% sucrose solution made in molecular grade water, at a final concentration of 30μg/ml, and sandwiched between two sterile layers of Parafilm M^®^. Whiteflies were given a six d feeding access to the sucrose solution containing dsRNA. Negative experimental controls consisted of 20% sucrose solution (1ml) containing 20μl dsRNA synthesis buffer and a 338 bp dsRNA homologous to *red-shifted green fluorescent protein* (*rsGFP*) (GenBank Accession number U70496.1), included as the non-homologous whitefly gene control.

The stability of dsRNA was confirmed before and after whitefly ingestion. The pre-ingestion conformation consisted of agarose (1.5%) electrophoresis of an aliquot of dsRNA as described above, to the expected dsRNA migration position, relative to the small molecular weight marker. Post-feeding confirmation was assessed by collecting a 2μl aliquot of sucrose solution from each chamber at 24 hr intervals for six d, and subjected to RT-PCR amplification with the respective dsRNA primers (Table 1) using the Superscript^®^ III One-Step RT-PCR System (Invitrogen, USA). Synthesis of cDNA was carried out at 55°C for 30 min, with 40 additional cycles at 94°C for 15 sec, 58°C for 30 sec, and 68°C for 1min. The expected size product for each was confirmed by agarose gel (1.5%) electrophoresis (data not shown). Amplicons were cloned and subjected to confirmatory DNA sequencing, as described above.

### Gene-specific bioassays

#### Neurotransmission bioassay

The *nicotinic acetylcholine receptor subunit alpha* (KF377802.1) gene was selected based on predicted sensitivity to the neonicotinoid insecticide, *I MaxxPro*^®^ (Univar^®^, Austin, TX, USA). To determine the LC_50_ adult whiteflies were treated with 10μg, 1μg, and 0.1μg/ml of *I MaxxPro*^®^. The LC_50_, herein determined as 10μg/ml for a 96 h exposure time [62], was used for the leaf disc bioassays [63]. Leaf discs were prepared by cutting a 60 mm circular shape from detached leaves of 8–10 leaf-stage cotton plant with a plastic 60 mm petri dish surface sterilized with 70% alcohol and allowed to air dry between each cut. Leaf discs were submerged in 10μg/ml *I MaxxPro*^®^ for 15 to 20 sec, air-dried, and placed, abaxial side up on a 2% agar in a 60 mm petri plate. For each replicated experiment, 200 adult whiteflies were collected using a simple hand held aspirator into a sample vial, transferred to a petri dish containing cotton leaf discs, and allowed to feed for up to six d, or until 100% mortality occurred. For RNAi analysis, 200 adult whiteflies were allowed feeding access to a 20% sucrose solution containing 30μg/ml dsRNA for 24 h and four and six d, prior to insecticide exposure, post-ingestion. The positive control consisted of adult whiteflies exposed to leaf discs coated with 10μg/ml solution of *I MaxxPro*^®^ with no RNAi treatment. The negative control consisted of adult whiteflies given access to insecticide-free leaf discs, as described above, and transferred to vials containing 20% sucrose in dsRNA buffer for a 24 h ingestion-access period.

#### Osmoregulation

The gut *AQP1* and *AGLU1* gut genes have a predicted role in osmoregulation. To determine the optimal osmolarity for the osmoregulation bioassay, adult whiteflies were allowed to ingest a range of molarities reported for plant sap, at 0.15M to 0.73M [41]. Osmotic tolerance was determined by exposing ~100 adults to 0.58 (20% w/v), 1.17 (40% w/v), or 1.75 (60% w/v) osm/l delivered through a Parafilm M^®^ membrane for up to six d. Since sucrose does not dissociate into ions, molarity of the solution becomes the osmolarity of the solution. The optimal whitefly survival, at 90%, was documented for 20% sucrose. Whiteflies were given a 24 h ingestion-access to 30μg/ml dsRNA in 20% sucrose homologous to gut genes, *AQP1* and *AGLU1*, respectively.

#### Thermotolerance

Heat shock proteins can confer heat tolerance in insects, including *B tabaci* [31, 64]. To determine the optimal conditions for the heat shock bioassay, adult whiteflies were exposed to 24, 28, 32, and 37°C and mortality was recorded every 24 h for six d. Whiteflies survived at 37°C for 24 h, and >24 h using the other three temperatures tested. Based on these results, 37°C was selected for the heat stress bioassay in this study. Whiteflies (200) were given ingestion-access to *hsp70* dsRNA for 24 h for four and six d, incubated at 37°C for 24 h and returned to 26°C. Mortality was recorded hourly for 24 h and at 24 h intervals thereafter for six d. The negative experimental control consisted of whiteflies given ingestion-access to 20% sucrose solution using the regime described for the RNAi experiment.

#### Sugar metabolism and transport

The gut genes, the *Tret-1* and the *Tre1*, are required for sugar metabolism and sugar transport out of the gut, respectively. The functional bioassay for these genes was conducted using 150 adult whiteflies given ingestion-access to dsRNA in 20% sucrose. Mortality was tabulated at 24 h and then on at 24 h intervals for four d and six d.

### Statistical analysis

Mortality studies consisting of two biological replicates and two technical replicates each were analyzed using the Student`s *t-test*, *p*-value <0.05. The results of whiteflies treated with dsRNA compared to the buffer control were considered significant when the *t*-stat value was greater than the *t*-critical value and the *p*-value was < 0.05 (Microsoft EXCEL for Windows, version 14.0.4734.1000). There was no significant difference between the buffer control and the non-homologous *rsGFP* negative controls, respectively.

### Quantitative PCR analysis of knock down

Whiteflies, post-dsRNA ingestion were held at -80°C for 15 min to slow metabolism and then transferred to a nuclease-free 1.5ml microfuge tube, held at -80°C. The total RNA was purified using RNeasy^®^ Plant Mini Kit (Qiagen Sciences, Maryland, USA), according to manufacturer’s instructions. The DNA concentration was determined using a Nanodrop 2000 spectrophotometer, prior to storage at -80°C. Approximately 0.5μg DNase I-treated (DNA-Free^™^ kit, Ambion, Austin, TX, USA) purified RNA was used for cDNA synthesis, using the High Capacity cDNA Reverse Transcription Kit (Applied Biosystems, CA, USA). The cDNA was diluted 1:10 and used for the qPCR analysis.

Whitefly gene expression was quantified using TaqMan qPCR at four and six d after ingestion-access, primer and probe sets (Table 2) for which were designed using the software available on line at: http://biosearchtech.com/realtimedesign-software-access.aspx. Whiteflies were collected from the feeding chamber one, four and six d post-treatment and subjected to qPCR amplification for *Tret1*. Results for *Tre1* quantification were obtained at four and six d only (because of undetermined cycle number for sample from d one consistently). The qPCR cycling conditions were: 50°C for 2 min, 95°C for 10 min, and 40 cycles at 95°C for 15 sec, with a final extension at 60°C for 1 min. Experiment consisted of two biological and three technical replicates, respectively, for each gene. The baseline gene used to calculate relative quantitative expression levels was the whitefly 18S rRNA gene transcripts.

**Table 2:** Gene-specific quantitative PCR primer-probe combinations.

Gene Name	Forward Primer	Reverse Primer	Probe
***AChRα***	TCGTACGGCCAGTAAGTGA	CGACGGGTCCGTGTTGTG	TCCAGGGTGCAGAACACGGT
***AGLU1***	CACCGCGTCGAACCTCATG	GCGAAGAGTTGGTTCAAGAGATG	CACTCCGCGCTCCAACCAGAAC
***AQP1***	CCTGCATTCGTCAGTGGAATTTG	GCAGTGACTCCACCGAGTATT	AAACATTGGGTGTACTGGGCCGGTCC
***Hsp70***	GGGGGCTTTTTGGATACCTTCTA	CCACAGAAGACAACCAGGATGG	AGGCTACTGCACGCTCACCTTCA
***Tre1***	GACGGACAGAGCAGAGTAGAC	TGCGGTGCTTTCCTGTAAC	TCTACGCATCCAACTTGACTCCAC
***Tret1***	GTGCCTCTTCTCTGGGCTTT	CACGTCGCAGTCCAGCTTC	TCGAACTCGTGGCTGCCTTCTA
***18S rRNA***	GACCGGAGCTTGCAATTGTTC	ATCGCCGCGAGGTTATGAC	CCGCGAACGAGGAATTCCCAGTA
***rsGFP***	CGGCCACAAGTTGGAATACAAC	TGAACGCTTCCATCTTCAATGTTG	CAACTCCCACAACGTATACATCACGGC

The ΔΔCt program (Applied Biosystems StepOne Plus instrument, Applied Biosystems, USA) was used to quantify transcript expression levels, with respect to baseline expression levels for a 70 bp fragment of whitefly ‘B’ biotype 18S ribosomal rRNA gene (Genbank Accession Z15051.1) (Table 2), which has been reported in insects to have relatively stable expression [65]. The delta Ct (ddCt) method [66], was used to plot fold-change in expression in relation to the negative controls, consisting either of whiteflies given ingestion-access on bioassay buffer and then subjected to the respective gene-specific bioassay, or adult whiteflies given ingestion-access to buffer only. The Student`s *t-test*, used to determine statistical significance, was implemented with a *p* value of <0.05) (Microsoft EXCEL for Windows, version 14.0.4734.1000).

## Results

### Gene knock down and whitefly mortality

#### Neurotransmission

**Fig 1:**
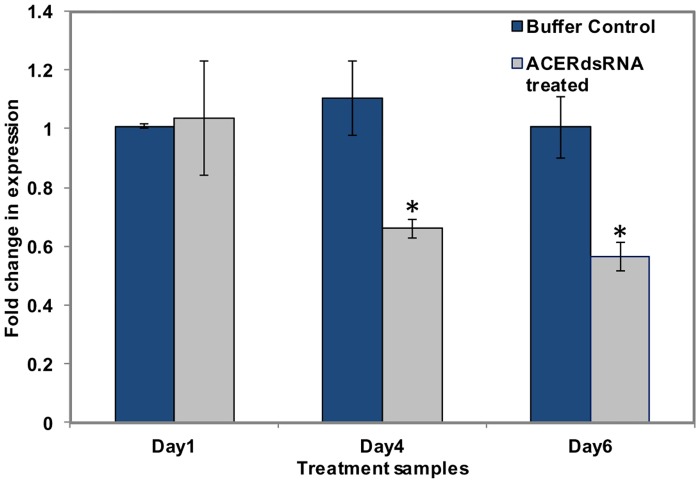
Results of real-time qPCR analysis of a neurotransmission gene target. The qPCR expression of target whitefly genes involved in neurotransmission, post-RNAi knock down. (A) The bar graphs show the comparisons in fold-change of expression of the *Acetylcholine receptor subunit alpha* (*AChRα*) when whiteflies were treated with 30μg/ml of *AChRα* dsRNA (gray bars), and the buffer treated control (blue bars) at day one, four, and six (x-axis). The “asterisk” indicates a significant *p-*value (< 0.05) (*t-*test).

Whitefly adults that were given ingestion-access to *AChRα* dsRNA, showed 40 and 50% reduced gene expression, at four and six d, compared to respective buffer control (Fig 1), and 45% mortality, which was significantly greater than buffer and non-homologous *rsGFP* controls, respectively, at 28% for both controls (Table 3 and S1 Table).

**Table 3:** Percentage mortality, post- *AChRα* dsRNA knockdown.

Treatment period	Buffer control	*rsGFP* control	*GOI *AChRα*
**1 d**	07.23	07.94	08.83
**4 d**	16.75	23.50	27.90
**6 d**	28.05	28.83	45.65

* GOI = Gene of interest.

The somewhat low percent mortality for whiteflies post-RNAi knockdown of *AChRα* suggested that gene silencing only minimally affected whitefly survival. For the insecticide bioassay, which was carried out post-dsRNA ingestion, whiteflies were exposed to *AChRα* dsRNA for one, four d or six d. Whiteflies were then given continuous exposure to *I MaxxPro*^®^-treated leaf discs for six days, and mortality was recorded daily. Mortality of insecticide-exposed whiteflies (Table 4) was three to four fold times greater compared to those that ingested buffer only, as the negative control. Time course results indicated that mortality was significantly different at 8, 10, and 12 h post-treatment, with the highest mortality at >90% six d post-ingestion. By day six, and 10 h-post-exposure to insecticide, >90% of dsRNA/insecticide-treated whiteflies had died, compared to buffer-only and buffer with insecticide negative controls, having significantly lower mortality, at 34% and 6%, respectively (S2 Table).

**Table 4:** Percent mortality post-*AChRα* dsRNA knock down, and exposure to *I MaxxPro*^®^^&^.

Treatment duration	RNAi 1 d mortality(%)			RNAi 4 d mortality (%)			RNAi 6 d mortality (%)		
*GOI -*AChRα*	RI**	BI^+^	UB^++^	RI	BI	UB	RI	BI	UB
**2h**	8.6	3.7	0.0	22.4	6.1	0.0	44.5	5.7	0.0
**4h**	12.9	7.0	0.0	25.4	9.1	0.0	57.9	12.3	0.7
**6h**	19.3	10.8	0.9	30.6	16.1	0.0	68.8	19.2	1.4
**8h**	23.2	17.7	1.9	41.1	19.1	0.3	83.8	26.0	5.3
**10h**	27.7	22.5	2.8	51	27.4	2.1	91.7	34.8	6.8
**12h**	35.6	26.6	3.5	64.3	35.4	5.1			
**2d**	51.1	33.2	8.2	79.8	54.3	12.5			
**3d**	61.6	38.3	13.2	90	59.8	19.6			
**4d**	69.8	45.7	17.8						
**5d**	85.5	51.8	23.7						
**6d**	97.3	58.3	29.7						

* GOI = Gene of interest

^&^ *I MaxxPro*^®^, Univar^®^, Austin, USA for acetylcholine receptor

** RNAi and insecticide treatment

^+^ Buffer and insecticide treatment

^++^ Untreated, buffer control

#### Osmoregulation

**Fig 2:**
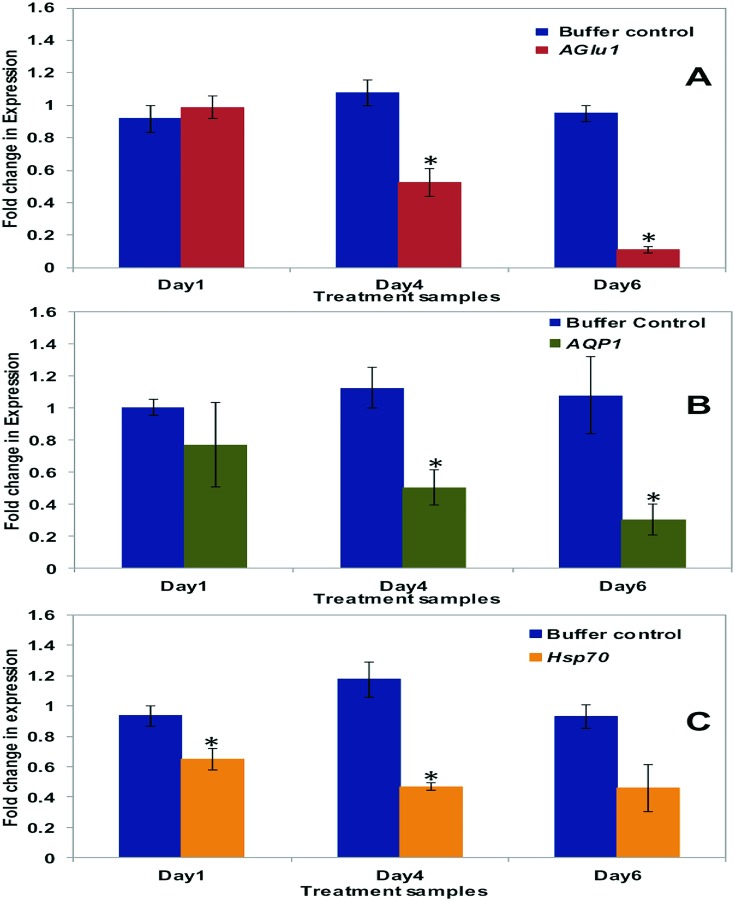
Results of qPCR analysis of gene targets involved in osmoregulation and thermo tolerance. Real-time qPCR amplification of expressed whitefly genes involved in osmoregulation, post-RNAi knock down. (A) Comparison of fold-changes in expression of *Alpha glucosidase1* (*AGLU1*) for whitefly treated with 30 μg/ml of *AGLU1* dsRNA (red) and buffer control (blue) at day one, four, and six, post-treatment (x-axis). (B) Comparison of fold-changes in expression of *Aquaporin1* (*AQP1*) for whiteflies treated with 30μg/ml of *AQP1* dsRNA (green), and the buffer treated control (blue) at day one, four and six, post-treatment (x-axis). (C) Comparison of fold-changes in expression of *Heat shock protein 70* (*Hsp70*) for whiteflies treated with 30μg/ml of *Hsp70* dsRNA (yellow), and the buffer control (blue bars) at day one, four, and six (x-axis). Of the three gene targets, *AGLU1* showed the most significant decrease in gene expression, which occurred on day six. The “asterisk” indicates a significant *p-*value (< 0.05) (*t*-test).

Post-ingestion, *AGLU1* and *AQP1* dsRNA treated whiteflies showed significantly reduced expression four and six d post-treatment, compared to buffer and *rsGFP* negative controls, respectively. Compared to negative controls, at four and six days post-dsRNA treatment, expression of *AGLU1* (Fig 2A) was reduced by ~50 and 80%, respectively, whereas, *AQP1* was ~50 and 70% less at four and six days post-dsRNA treatment (Fig 2B). At six d post-dsRNA treatment, mortality for *AQP1* dsRNA and buffer only treated whiteflies was at 84% and 28%, respectively. Similarly, at six days post-treatment, mortality of whiteflies treated with *AGLU1* dsRNA was significantly different, at 92%, compared to the buffer and non-homologous *rsGFP* controls, at 28% each, respectively (Table 5 and S1 Table).

**Table 5:** Percent mortality post- *AQP1* and *AGLU1* dsRNA knockdown.

Treatment duration	Buffer Control	rsGFP control	* GOI-*AQP1*	* GOI-*AGLU1*
**1 d**	07.23	07.94	26.08	25.58
**4 d**	16.75	23.50	67.83	69.42
**6 d**	28.05	28.83	84.33	91.92

* GOI = Gene of interest.

#### Thermotolerance

Knockdown of *Hsp70* resulted in ~30% significantly reduced mRNA levels on d one, and by d four and six, *Hsp70* expression was decreased by ~50%, (Fig 2C), compared to the buffer control. Mortality of whiteflies post-*Hsp70* dsRNA ingestion was 35%, greater than for buffer and non-homologous *rsGFP* controls, at 28% each, respectively. (Table 6 and S1 Table).

**Table 6:** Percent mortality for *Hsp70*-dsRNA knockdown.

Treatment Duration	Buffer Control	rsGFP control	* GOI- *Hsp70*
**1 d**	07.23	07.94	07.05
**4 d**	16.75	23.50	21.15
**6 d**	28.05	28.83	34.55

* GOI = Gene of interest.

Results of the heat tolerance bioassay indicated that whiteflies were increasingly sensitive to *Hsp70* knock down, when exposed to successively higher temperatures. At 37°C, mortality of whiteflies exposed to *Hsp70* dsRNA for 24 h reached 100% by day six, compared to the buffer control at 34%. Whiteflies given a four d ingestion-access to *Hsp70-*dsRNA, and then given a 37°C heat treatment, exhibited 100% mortality three d post-treatment, compared to 27% for buffer-control whiteflies. Heat stress at 37°C following a six d *Hsp70* dsRNA exposure, resulted in 100% whitefly mortality by 10 h, compared to 15% for buffer control (Table 7 and S2 Table).

**Table 7:** Percent mortality post-*Hsp70* knock down at 37°C.

Treatment duration	RNAi 1 d mortality (%)		RNAi 4 d mortality (%)		RNAi 6 d mortality (%)	
Hsp70	RT***	BT^+++^	RT	BT	RT	BT
**2h**	3.2	0.0	22.1	.25	39.6	1.5
**4h**	7.0	0.0	26.9	0.25	54.9	1.5
**6h**	11.8	0.5	32.6	0.5	77.5	6.6
**8h**	19.1	2.1	39.1	2.9	96.2	11.5
**10h**	24.4	4.6	49.0	8.1	100.0	14.8
**12h**	32.8	7.2	55.2	13.4		
**2d**	53.9	14.5	91.3	21.8		
**3d**	70.9	18.0	99.0	27.6		
**4d**	80.8	22.9				
**5d**	89.86	29.3				
**6d**	95.75	33.7				

*** RNAi and heat-stress treatment

^+++^ Untreated, buffer with temperature experimental control

#### Sugar metabolism and sugar transport

**Fig 3:**
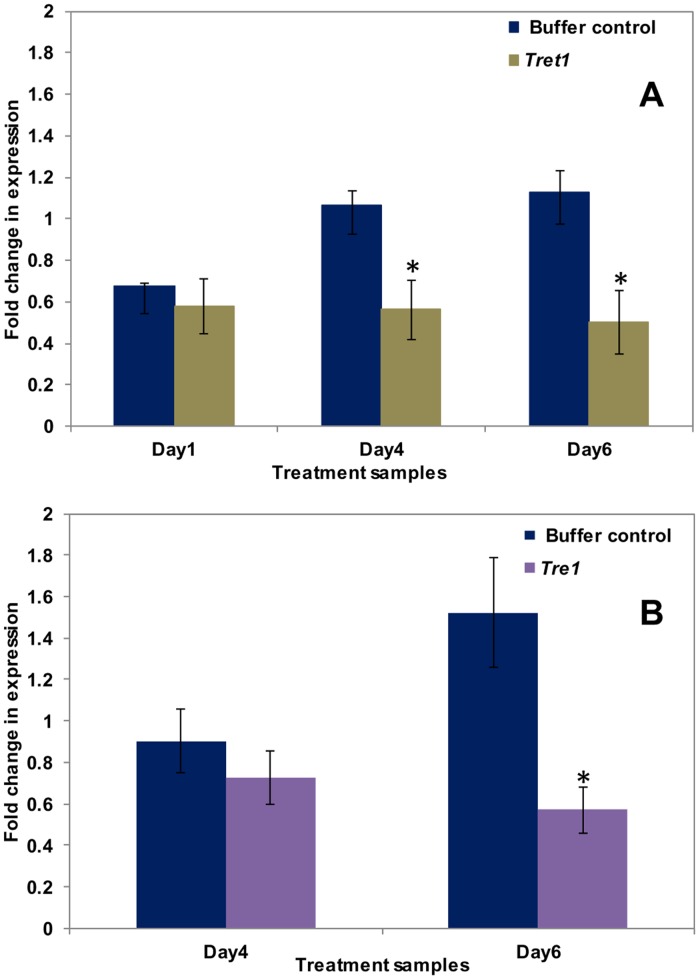
Results of qPCR analysis for gene targets involved in sugar metabolism and transport. The qPCR amplification of expressed whitefly genes involved in sugar metabolism and transport, post-RNAi knock down. (A) Comparison of fold-change expression of *Trehalose transporter 1* (*Tret1*) for whiteflies treated with 30μg/ml of *Tret1* dsRNA (tan), and the buffer control (blue bars) on day one, four, and six, post-treatment (x-axis). (B) Comparison of fold-change in expression of *Trehalase1* (*Tre1*) for whiteflies treated with 30μg/ml of *Tre1* dsRNA (purple) on day four and six (x-axis). Sugar metabolism genes were ~ 30–40% down regulated. The “asterisk” indicates a significant *p-*value (< 0.05) (*t*-test).

Whiteflies allowed to ingest *Tret-1* dsRNA had 50% reduced mRNA expression, four and six d, post-treatment compared to the buffer control (Fig 3A) whereas, whiteflies treated with *Tre1* dsRNA, showed ~45% reduced mRNA levels, by six d post-treatment (Fig 3B). Mortality of whiteflies exposed to *Tret-1* and *Tre1* dsRNA for one d, was at 9.6%, compared to the buffer and *rsGFP* controls, at 4.2 and 3.3%, respectively (Table 8). Percent mortality increased with longer exposure times, reaching 70.25 and 72.56% for *Tre1* and *Tret-1* dsRNA treatments, respectively, compared to 22.69 and 27.28% for buffer and *rsGFP* controls, respectively (Table 8 and S1 Table).

**Table 8:** Percent whitefly mortality for *Tre1* and *Tret1* knockdowns.

Treatment duration	Buffer Control	*rsGFP* control	* GOI- *Tre1*	* GOI- *Tret1*
**1 d**	04.20	03.30	09.60	09.60
**4 d**	13.30	14.51	52.15	55.13
**6 d**	22.69	27.28	70.25	72.56

* GOI = Gene of interest.

## Discussion

Phytophagous insects, including the whitefly *B tabaci*, cause direct feeding damage and transmit plant viruses, causing substantial losses in agriculture. Pesticides have been used to reduce whitefly population size to abate feeding damage and lower virus transmission rate [67]. Despite the use of transgenic plant technology e.g. *Bacillus thuringiensis* (Bt) toxin, to manage high-impact lepidopteran pests in cotton, maize, and soybean crops [68], a similar approach has not been pursued for controlling insects that have piercing-sucking mouthparts by which they feed on plant phloem and xylem. Such technology is expensive, relies on availability of species-specific targets, and requires extensive potential off-target effects to other hemipterans. Thus, use of dsRNA to induce RNA interference (RNAi), or knock down of gene expression, has become a promising alternative technology for insect control that does not require the *in planta* expression of dsRNA.

In this study RNAi was used to knock down the expression of six whitefly gut-specific candidate genes, *AChRα*, *AGLU1*, *AQP1*, *Hsp70*, *Tre1* and *Tret*. Targets were selected from a whitefly *B*. *tabaci* ‘B biotype’ gut library, based on their predicted involvement in neurotransmission, osmoregulation, thermotolerance, sugar metabolism, and sugar transport. The dsRNAs were designed to a unique region within each gene, and shared 100% sequence homology with the respective target. Whiteflies were given an ingestion-access period on synthetic dsRNA in 20% sucrose delivered through a parafilm membrane to simulate ingestion in plant sap during feeding.

Gene silencing was shown to result in significantly reduced gene expression for all six genes tested, at 70–75% mortality for *AGLU1*, *AQP1*, *Tre1*, and *Tret1*, one to six d, post-dsRNA ingestion-access, with ~70% knock down efficacy. Although *AChRα* and *Hsp70* knock down was significant, compared to the other four targets, mortality was only 45 and 35%, respectively. This observation is most probably explained by presence of parallel gene expression pathways for gut-expressed isoforms, or by homologous gene expression elsewhere in the whitefly body.

Nonetheless, silencing of *AChRα* and *Hsp70* targets resulted in high whitefly mortality of 80–90% only six d post-dsRNA ingestion. Similarly, whiteflies silenced for *Tret1* and *Tre1* expression, when deprived owing to silencing of proteins that function in sugar transport and sugar metabolism, respectively, exhibited significant mortality, e.g. 75–85%.

In this study, functionally-specific bioassays were carried out to guage whitefly responsiveness to a respective treatment, administered post-dsRNA ingestion and RNAi, intended to challenge functionality of the silenced gene and resulted in altered survivability. For example, whiteflies exposed to heat shock or a neonicotinoid insecticide treatment showed two-fold greater mortality as a result of compromised induction of proteins that were otherwise expected to confer protection to heat stress or detoxify neonicotinoid insecticides. In these examples, whiteflies were rendered ‘unprotected’ as a direct result of RNAi, providing robust support that dsRNA-mediated silencing results in the effective knock down of gut genes and compromised whitefly fitness. Consequently, under heat stress, hypothetically, insecticide efficacy could be greatly enhanced at lower-than-expected insecticidal rates.

Imidacloprid-resistant *Nilaparvata lugens*, which incurred mutations in the neonicotinoid receptor gene *AChRα* [69], showed reduced mRNA expression of the receptor as well as increased neonicotinoid susceptibility [70]. Similarly, *AChRα* silenced whiteflies tested here were icreasingly susceptible with increased exposure to the neonicotinoid insecticide, compared to the pre-challenge mortality. This phenomenon can most likely be explained by the continuous disruption of neurotransmission, the latter being the mode of action for this class of insecticides, which by design leads to rapid death. In this study, whitefly sensitivity to *I MaxxPro*^®^ increased with RNAi progression and is consistent with the initially low mortality (at 35%) shortly after ingestion, and dramatic increase in death rate, at >70% six-d post-dsRNA ingestion. In contrast to *N*. *lugens* for which target site sensitivity to imidacloprid has been reported, resistance to the neonicotinoid in *B*. *tabaci* ‘B biotype’ is caused by impaired monooxygenase activity, an enzyme required for detoxification [71]. The prolonged use of neonicotinoids for *B*. *tabaci* management has often resulted in pest control failure [72]. Based on the results herein, RNAi has great potential to remedy the above scenario by enhancing susceptibility in *B*. *tabaci* to imidacloprid, while also slowing development of resistance by reducing both, application rates treatment number to achieve effective control.

Importantly, whitefly gut-directed RNAi induction of post-transcriptional gene silencing by oral delivery of dsRNA *de facto* provides support for the predicted or *‘in silico’* functional assignments for all six gut-specific candidates evaluated here. These results complement those reported in a previous study demonstrating systemic allelic silencing for exclusively, *in silico*-identified genes [73]. These results unequivocally demonstrate the advantage of dsRNA-targeting to the gut, which dramatically affects local, organ-specific gene expression there. Since gut is the first point of contact by compounds ingested in plant sap during whitefly feeding, targeting them specifically is expected to have a nearly immediate affect on functional responses that may be critical for fitness and/or survival.

Evidence that the *B*. *tabaci* genome encodes *sid-1* and additional genes known to function in the systemic RNA silencing pathway, predicts the potential for amplification of the silencing signal and knock down of global expression, following down-regulation of the primary gene target [12, 74], a phenomenon reported in the cotton pest *Cylas punticollis* [75]. In the whitefly, the rapid appearance of a ‘response’ that lead to a measurable phenotype e.g. mortality or prolonged survival post-treatment suggests that both local and global silencing are responsible for the perturbation of gene function and the associated phenotypic responses.

Knowledge of conserved, predicted functional domains, or of other parts of coding regions that share high sequence homology across different insect species or genera that colonize the same range of host plants, could make it possible to target more than one insect species with the same dsRNA. Consequently, down-regulating expression of divergent gene targets with a mixture of unique dsRNAs could provide a means of achieving broad-spectrum protection, with minimal off-target effects. Lastly, additive and/or synergistic affects would be expected to result from simultaneously silencing multiple gut gene targets, particularly, when at least some are essential for combatting environmental stress.

For those whiteflies that are vectors of plant viruses, reducing the population size is well known to contribute towards lowered transmission frequency. Thus, identification of genes required for vector-mediated virus transmission that are specifically expressed in the gut, for example, those essential for virion translocation across the gut membrane barrier, or that serve as receptors used by the virus to enter the salivary glands, could open possibilities for dsRNA-mediated disruption of virus transmission, and thereby to a previously unavailable tool for virus disease management.

The ability to deliver effective dsRNAs to plants through root or foliar uptake has great promise for protecting plants from phloem and leaf-feeding insects [36,76–78]. Genes expressed in the gut are among the most likely to be rapidly silenced and at high efficacy because of their vulnerability to the affects of compounds or small molecules inherent in plant sap, which is ingested by these insects in large quantities during feeding. The dsRNA hairpins expressed in transgenic-plants that share homology with the respective target gene, have already been shown to induce gene silencing in hemipteran insects, including the aphid, *M*. *persicae*, whitefly, *B*. *tabaci*, and leafhopper, *N*. *lugen*s, in both laboratory and field studies [79–81]. At present, transgenic solutions are not economical for use in non-agronomic (large acreage) crops because many varieties are required to accommodate widely variable environments and market needs, and so are produced on relatively small-scale, compared to agronomic crops. The practical implementation of RNAi as an alternative to traditional pesticides for crop protection [33, 34, 36 and 37] will require further studies to determine the specific dsRNA application rates, as well as persistence of dsRNA in the plant host and subsequently, the insect targets. Other important considerations will be environmental safety and potential for non-target and off-target effects.

## Conclusions

The dsRNA-mediated RNAi knock down efficacy was not 100% for any of the six gut gene targets tested, however, substantial down-regulation of gene expression was sufficient to achieve significant whitefly mortality ranging from 35–90%. Even so, at these rates of knock down, albeit under laboratory conditions, whitefly ingestion of dsRNAs with 100% homology to whitefly gut gene sequences has promise for insect management under greenhouse and field conditions. The RNAi-gene-specific bioassays used to validate knock downs, were designed to result in altered survivability based on predicted gene function. Similarly strategic bioassay approach can be designed for quantifying or qualifying other phenotypic responses in insects, such as, fecundity, stress response, or virus transmissibility. The evidence provided for rapid response, e.g. within hours or a few days, and significant knock down efficacies for all the six *in silico*-predicted gene targets, supports the prediction that they are expressed in the whitefly gut. In addition, at least one of the gene targets is also expressed in non-gut tissue based on the apparent ‘rescue’ from gene silencing, a phenomenon known to occur in more than one cell, tissue or organ type. Future studies are expected to aid in an improved understanding of organ/tissue-specific, and body-wide expression, and in synergism and/or compensatory expression scenarios, such as those reported here. In those instances when knock down was less robust than anticipated e.g. for *AChRα and Hsp70*, partial silencing can likely be attributed to compensatory expression of a homolog in the gut or elsewhere in the whitefly body. For the *AChRα* knock down whiteflies, sustained insecticide exposure led to increased sensitivity, and a lower effective dose was required to achieve mortality, suggesting that the *AChRα* receptor was progressively saturated at an increasingly lower insecticide dose, as silencing progressed. The implications associated with deployment of dsRNAs reported here for field use are several-fold. First, *AChRα*-dsRNA has potential for lowering the effective rate and number of insecticide applications, after an initial treatment essential for initiating RNAi. Fewer insecticide applications therefore would be expected to slow development of insecticide resistance. Second, *Hsp70* knock down rendered whitefly more susceptible to heat, post-dsRNA ingestion, and consequently, has great promise for enhancing whitefly control in environments where *Hsp70* up-regulation would be essential for heat protection. Lastly, the ‘B’ biotype/cryptic species group is endemic to northern Africa and the Middle East [1, 2] where extreme temperatures prevail. This study provides evidence that ‘B biotype’ fitness was rapidly and substantially reduced owing to RNAi, and further suggests that the susceptibility to heat stress by less-heat resilient cryptic species, such as the Q biotype, endemic to the Mediterranean region [1], would be even greater, when subjected to *Hsp70*-dsRNA-mediated knock down.

## Supporting Information

S1 Table Results of RNA interference mortality assays. (XLSX)

S2 Table Results of gene-specific post dsRNA-treated bioassays. (XLSX)
